# Virtual-source imaging and repeatability for complex near surface

**DOI:** 10.1038/s41598-019-53146-w

**Published:** 2019-11-13

**Authors:** Yang Zhao, Tao Liu, Genyang Tang, Houzhu Zhang, Madhumita Sengupta

**Affiliations:** 1 State Key Laboratory of Petroleum Resource and Prospecting, and Unconventional Petroleum Research Institute, China University of Petroleum, 18 Fuxue Road, Changping District, Beijing, 102249 China; 2 Sinopec Petroleum exploration & Production Research Institute, 100083 Beijing, China; 3 Houston Research Center, Aramco Services Company, Houston, TX 77074 USA

**Keywords:** Geophysics, Seismology

## Abstract

Based on seismic interferometry, the virtual source (VS) method is able to produce virtual gathers at buried receiver locations by crosscorrelating the direct-downgoing waves with corresponding reflected-upgoing waves from surface-source gathers. Theoretically, the VS records can improve seismic quality with less negative impact from overburdened complexities. However, shallow complex structures and weathering layers at near surface not only severely distort the wavepaths, but also introduce multiples, surface waves, scattering noise, and interference among different wave modes. These additional seismic responsescontaminate both direct-downgoing and reflected-upgoing wavefields. As a result, the VS gathers experience spurious events and unbalanced illuminations associated with distorted radiation patterns. Conventional stacking operator can produce significant artifacts for sources associated with ineffective-wavepath cancellation. We review three publications and summarize a comprehensive workflow to address these issues using data-driven offset stacking, wavelet-crosscorrelation filtering, and radiation-pattern correction. A data-driven offset stacking theme, with each individual source contribution is weighted by certain quality measures, is applied for available offsets. The wavelet crosscorrelation transforms time-offset data into local time-frequency and local time-frequency-wavenumber domains. Filters are designed for the power-spectrum in each domain. The radiation-pattern correction spatially alters the contaminated direct-wavefields using a zero-phase matched filter, such that the filtered wavefield is consistent with the model-based direct P-wavefields observed at buried receiver locations. Our proposed workflow produces significant improvement as demonstrated in the 13 time-lapse field surveys that included substantial repeatability problems across a 17-month survey gap.

## Introduction

Thirteen repeated 2D surveys were acquired over the course of 19 months above an onshore field in the Middle East. The first six surveys (S1-S6) were collected within a period of three-months, after a 17-month break, an additional seven surveys (S7-S13) were acquired over a period of a week. All surveys were acquired with Mertz 26 vibrators with most of the shot locations repeated with better than 1 m accuracy. A 2D line of 80 receiver stations were installed with geophones cemented in individual vertical boreholes with 30 m spacing. The sensors are at depths of 50 m below surface. Dense 3D areal shooting (7.5 m inline and 7.5 m crossline) was performed for linear noise removal, and above the output VS location for optimum illumination. The corresponding offsets range between 0–2400 m. The geology is composed of a number of layers with large velocity contrasts overlaying a target reflector at approximately 2000 m depth. The thickness of superficial sand-layer varies from a few meters to more than 50 meters. The near-surface that is covered by thick sand is considered to be an area which produces suboptimal seismic data quality. Below the near-surface, there is a simple layer-cake geology and the associated reflector dip is less than 5 degrees. According to Bakulin, *et al*.^1^, the image quality and repeatability of both post-stack and pre-stack data suggests the majority of the changes are associated with extremely shallow near-surface variations. The use of a buried system provides an opportunity to redatum the surface sources to the receiver level using the virtual-source methods. This is expected to greatly improve the image quality, coupling changes, and diurnal/seasonal temperature variations in the time-lapse processing.

The virtual-source (VS) method, based on seismic interferometry, originates from global seismology. Shapiro and Campillo^2^ and Lobkis and Weaver^3^ extracted Green’s functions by crosscorrelating long sequences of ambient seismic noise, normally discarded in traditional processing, which contains information about the structure of the shallow and middle crust. In exploration seismology, the VS may help produce images from underneath a complex overburden with without knowledge of the overburden velocities and near-surface changes. This workflow crosscorrelates direct-downgoing waves with upgoing (reflected) seismic waves to retrieve the reflection response, and then redatums the surface-source records to buried receiver locations^4,5^. The power of actual buried receivers is that they allow for the direct measurement of wavefields propagating through the intricate overburden structure, and offers much more accurate measurements than model-based approaches^6,7^. Wapenaar^8^ and van der Neut, *et al*.^9^ derived interferometric representations to retrieve exact VS Green’s function in elastic media. More importantly, redatuming is able to improve time-lapse survey repeatability by correcting time-lapse noise, such as that induced by near-surface diurnal and seasonal weather cycles as well as small changes in acquisition geometry and shot coupling^5^. In summary, VS should simplify a recorded seismic wavefield and correct the distortions associated with heterogeneities located between the seismic sources and the receivers.

The VS theory^10–12^ states that a redatumed VS response can be fully recovered by integration over sources located on a closed surface surrounding the receiver. In the stationary-phase zone, contributions to the VS are summed constructively andthe common ray paths can be effectively cancelled. However, this is not true for sources located outside of the stationary zone^13–15^. The current practice of finding source contributions within the stationary phase zone is based on a simple distance-weighting scheme^5^. Near offsets (with respect to the VS) are usually considered to be stationary, leading to a simple linear weight function and decays exponentially with offets. Nevertheless, due to the limited offsets and insufficient sampling of land acquisition data, repeatability and image-quality issues still exist because the VS assumptions failed^8,16^. As a result, onshore seismic data processing requires specialized denoising steps to restore the coverage so that redatuming can work effectively. For example, wavefield separation (dual sensors) and direct-arrival windowing have been shown to be the most effective approaches to isolate the direct-downgoing P-wave^5,17,18^. However, the complex near surface can severely distort the ray paths so that even near-offset sources may lie outside of the stationary area, whereas far-offset sources may provide constructive contributions to VS. Additionally, near-surface reverberations, surface multiples, and other mode-converted waves may leak into the windowed early arrivals and further corrupt the direct wavefields^19,20^. As a result, early arrivals often contain near-surface reverberations and multiples which interfere with upgoing reflections. Wavefield contamination results in poor suppression of multiples, scattered energy, and surface waves. The amplitude radiation-pattern of the VS distorts and creates non-uniform illumination for target reserviors. This leads to degraded image quality where the VS stack has a signal-to-noise ratio (S/N) lower than that obtained by non-VS processing.

We experienced the abovementioned near-surface challenges in this time-lapse monitoring feasibility dataset where the spurious events remain as key issues in the VS processing. To address these challenges, we propose a new VS redatuming workflow to improve the processing quality in the steps of traditionalprocessing: offset stacking, crosscorrelation, and unbalanced illumination. During offset stacking, we developed a data-driven method taking into account the non-stationary behavior via a selective weighting scheme over varying offsets. We adaptively adjust the stacking weights to honor stationary-source contributions andsuppressing the non-stationary effects. In terms of crosscorrelation, we perform VS in the wavelet-wavenumber domain to exploit the non-stationary characteristics of severe noise effects. More specifically, this step maps data from the time-offset (TX) domain into the time-frequency (TF) or time-frequency-wavenumber (TFK) domain in which the signal and noise can be better separated. The original signal phase is preserved, but the amplitudes are filtered to suppress VS spurious events. This method allows effective noise suppression and high-resolution separation of scattered energy and surface waves using TF or TFK filtering of the wavelet-correlation coefficients. We then construct and apply a matched filter iteratively to correct and recover the 3D amplitude radiation pattern for each buried receiver. Specifically, this matched filter is designed to involve the 3D FK amplitude spectrum of the direct arrivals for each VS, estimating the ideal spectrum of the direct P-wave by approximating the near surface with a homogenous model, and iteratively solving for the filter coefficients to minimize the misfit function of the radiation pattern between the computed and the ideal spectrum. Next, the P-wave velocity for the local homogeneous model is updated. After the matched filter, the output VS, is expected to possess isotropic radiation patterns and provide balanced illuminations. These renovated steps compensate for near-surface complexity and reduce time-lapse noise, and therefore produces redatumed data with fewer artifacts. The output VS records are then stacked to produce final image.

The proposed VS workflow consists of the following steps: (a.) estimate and update a 3D matched filter; (b.) applying the 3D matched filter to the direct-arrival wavefields; (c.) store the corrected direct arrivals if misfit criteria is satisfied; (d.) transform direct arrivals and reflections into the wavelet domain; (e.) crosscorrelate the wavelet coefficients; (f.) denoise spurious events and suppress surface waves via TFK domain filtering; (g.) invert the wavelet-transform filtered data back to the TX domain; (h.) estimate the predicted upgoing wavefields and compute the cross-coherence weight with the original upgoing wavefields; (i.) data-driven offset stack the crosscorrelation gathers over the available offsets with the obtained weight. The output VS records are then stacked to produce a final image. A diagram summarizing the abovementioned workflows is given at the end. However, not all of the overburden related effects can be removed from the estimated reflectivity using the proposed workflow in which case deconvolution of full downgoing wavefields with sufficient wavefield separations^9^ is required.

## Review of the Conventional VS and the Challenges

To investigate the reasons of the degradation of the VS responses in the presence of complex near-surface, we briefly review the VS methodology here. It essentially involves crosscorrelating the seismic responses observed at different receivers to the same source and summing over the contributing sources, which can be written as^21^

1 $$V({r}_{B}|{r}_{A};t)=\sum _{{r}_{S}}g(r)D({r}_{A}|{r}_{S};t)\otimes U({r}_{B}|{r}_{S};t)$$

where ⊗ denotes crosscorrelation. *r*_*A*_, *r*_*B*_ and *r*_*S*_ denote the spatial locations of the two receivers at *A* and *B*, and at the source location *S*, respectively. *V*(*r*_*B*_|*r*_*A*_; *t*) is the resulting interferometric data with the time lag *t* at receiver *r*_*B*_ when *r*_*A*_ is treated as a virtual source. *D*(*r*_*A*_|*r*_*S*_; *t*) represent the direct-downgoing wavefields recorded at buried receivers *r*_*A*_, while *U*(*r*_*B*_|*r*_*S*_; *t*) represents the reflected-upgoing wavefields at *r*_*B*_. *g*(*r*) is the weight operator which is a simple Gaussian function centered at the VS location: $g(r)={e}^{-{r}^{2}/2{R}^{2}}/\sqrt{2\pi }R$, where *R* is the maximum offset, *r* is the offset between the source *r*_*S*_ and the buried receive*r r*_*A*_. *g*(*r*) ranges from 1 at the receiver location to 0 at the maximum offset.

**Figure 1:**
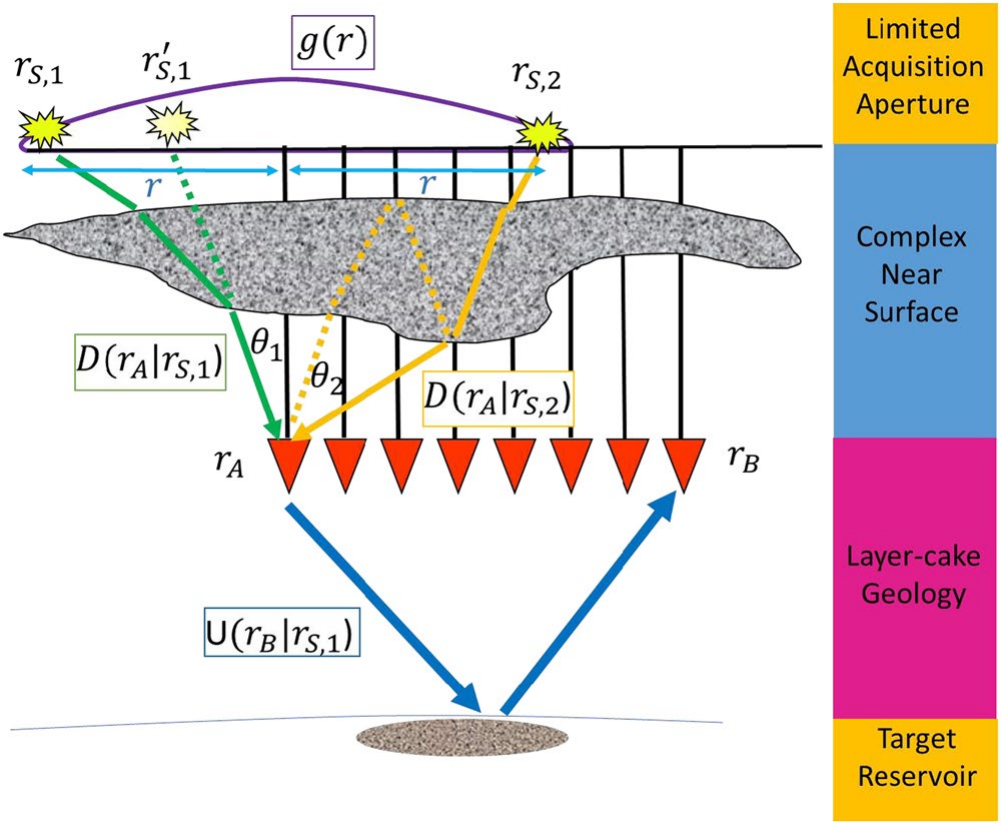
An illustration of virtual-source redatuming in the presence of near-surface complexity. Two surface sources, with the same distance (blue solid lines) with respect to the VS, generate two direct arrivals recorded by the buried receivers. The straight ray path is broken into a set of piecewise rays (yellow solid lines) and results in inequivalent angle coverage. The surface multiples, near-surface reverberations (yellow dashed lines) are also introduced to contaminate direct arrivals (yellow solid lines). The conventional stacking operator (Gaussian function *g*(*r*)) is shown as a purple curve on the top of surface. The surface sources *r*_*S*_ generates direct arrivals recorded by the buried receivers *r*_*A*_ to *r*_*B*_, which make a positive contribution to VS. The near-surface complexity breaks the raypath into a set of piecewise rays (green solid lines) with potentially complex raypaths. The incorrectly projecting source *r*_*S*′_ (green dashed lines) does not share a common raypath, and therefore makes a nonstationary contribution to the VS. Conventional weighting *g*(*r*) assigns inappropriate weights to surface sources and degrades VS responses.

**Figure 2:**
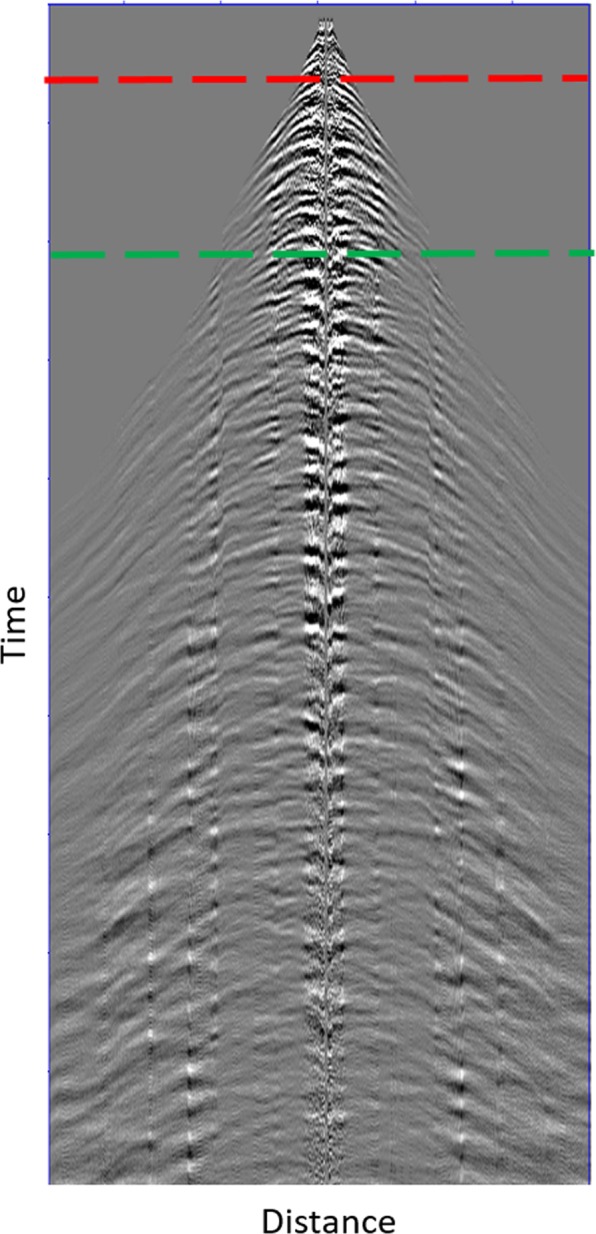
A geophone shot record (with source location located in the center of the array) after noise removal in the common-receiver domain. The red dashed line indicates the time window of direct arrivals, and the green dashed line is for the reflections.

Figure 1 summarizes underlying principle that the near-surface complexity degrades the VS response. Direct arrivals *D*(*r*_*A*_|*r*_*S*,1_) and *D*(*r*_*A*_|*r*_*S*,2_) excite from surface sources *r*_*S*,1_ and *r*_*S*,2_, and are recorded by a buried receiver *r*_*A*_. *r*_*S*,1_ and *r*_*S*,2_ that have the same offset *r*(light blue solid lines) with respect to *r*_*A*_. In the absence of the wavefield separation capability, we treat the direct arrivals as the downgoing wavefields and the late-arriving reflections as the upgoing wavefields, respectively. Figure 2 shows a typical record. We consider the unprocessed near-offset data as the downgoing wave, usually at offsets less than 30 m and within 100 ms (the time section above the red dash lines). These values were estimated from our experiences that the early arrivals are relatively unpolluted and is still dominated by the direct P-wave. The later-arriving reflections (>300 ms, the time section below the green dashed lines) at all offsets are used as approximations to the upgoing waves. The horizontal distance is 2400 m and the vertical time axis is from 0 s to 2 s (~3300 m).

There are mainly three challenges that need to be addressed:

1. Nonstationary offset stacking. Figure 1 illustrates a scenario in which *D*(*r*_*A*_|*r*_*S*,1_) and *U*(*r*_*B*_|*r*_*S*,1_) share a common raypath from shot *r*_*S*,1_.and will have the correct compensation for the phase associated with the near-surface propagation by crosscorrelating with *U*(*r*_*B*_|*r*_*S*,1_), and making a stationary contribution to *V*(*r*_*B*_|*r*_*A*_). Therefore, it should be added into *V*(*r*_*B*_|*r*_*A*_) with maximum weight. However, since the weight *g*(*r*) (purple bell curve) is not data driven, it provides a minor weight during offset stacking. In contrast to *r*_*S*,1_, the near-offset shot *r*_*S*,1′_, linear mapped from the buried VS to the surface location, does not share the common raypath (green dash lines) with *r*_*A*_ and therefore has deconstructive contributions. From the above analysis, we observe that weights *g*(*r*) based on the surface location (purple bell curve) could assign inappropriate weights and lead to non-stationary contributions. Other shots such as *r*_*S*,2_ may also qualify for contribution to *V*(*r*_*B*_|*r*_*A*_) constructively, as long as its source raypath to *r*_*A*_ can cancel with the raypath to *r*_*B*_ effectively.
2. Multi-wave mode contamination. The intricate overburden introduces near-surface reverberations (yellow dashed lines), and S-wave modes which contaminate direct arrivals (yellow solid lines) regardless of careful time-windowing and spatial energy tapering. Considering this, each of the up- and downgoing-wavefields can be written as a summation of different modes:
  2 $$\begin{array}{rcl}D({r}_{A}|{r}_{S};t) & = & {D}_{LP}({r}_{A}|{r}_{S};t)+{D}_{LM}({r}_{A}|{r}_{S};t)+{D}_{LS}({r}_{A}|{r}_{S};t)+\mathrm{...}\\ U({r}_{B}|{r}_{S};t) & = & {U}_{LP}({r}_{B}|{r}_{S};t)+{U}_{LM}({r}_{B}|{r}_{S};t)+{U}_{LS}({r}_{B}|{r}_{S};t)+\mathrm{...},\end{array}$$
  where subscripts *P*, *M*, *S* represent direct *P*-arrivals, near-surface multiples, and S-waves modes, respectively. We add subscripts *L*(land data) in front of *P*, *M*, *S* to avoid subscript conflictions in other sections. (*D*_*LP*_, *U*_*LP*_), (*D*_*LM*_, *U*_*LM*_), (*D*_*LS*_ and *U*_*LS*_) are the associated up- and downgoing components, respectively. Only the crosscorrelation of the downgoing *D*_*LP*_(*r*_*A*_|*r*_*S*_; *t*) with the upgoing *U*_*LP*_(*r*_*B*_|*r*_*S*_; *t*) in Eq. (2) would produce a kinematically correct event. Other terms, *D*_*i*_(*r*_*A*_|*r*_*S*_; *t*) * *U*_*j*_(*r*_*B*_|*r*_*S*_; *t*) where *i* is not the same as *j*, would typically generate spurious events.
3. Unbalanced illuminations. The straight ray path breaks into a set of piecewise rays (yellow and green solid lines). This generates an inequivalent angle coverages (*θ*_1_ of *D*(*r*_*A*_|*r*_*S*,1_) ≠ *θ*_2_ of *D*(*r*_*A*_|*r*_*S*,2_)), although *r*_*A*_ is covered by the same offset therefore the VS illumination is heavily biased beneath the overburden.

## Method: A Comprehensive VS Workflow to Tackle Complex Near-Surface

### Step 0: Review of Continuous wavelet transform

At the beginning of the methods section, we briefly review the fundamentals of continuous wavelet transformation (CWT) as it underpins the following sections heavily. The CWT is used to decompose a signal into wavelets. Wavelets are small oscillations that are highly localized in time. While the Fourier Transformation decomposes a signal into infinite length sines and cosines, it effectively loses all time-localization information. The CWT’s basic functions are scaled and shifted versions of the time-localized mother wavelet. The CWT is used to construct the TF representation of a signal and offers good time and frequency localization^22–28^. The CWT is an excellent tool for mapping changing properties of non-stationary seismic signals.

For a given 1D seismic trace *D*(*t*), the forward CWT expands it from 1D to a 2D signal:

3 $${\Psi }^{\phi }({f}_{\alpha },\tau )=\frac{1}{\sqrt{{f}_{\alpha }}}{\int }_{-\infty }^{\infty }D(t)\phi (\frac{t-\tau }{{f}_{\alpha }})dt,$$

where Ψ^*φ*^(*f*_*α*_, *τ*) is a 2D wavelet coefficient, and *φ*(*t*) denotes the Ricker source wavelet. The symbols *f*_*α*_ and *τ* are Ricker wavelet frequency and local time delay, respectively. The inverse wavelet transformation brings the 2D signal back to the time domain:

4 $$D(t)=\frac{1}{{C}_{\phi }}{\int }_{0}^{\infty }{\int }_{-\infty }^{\infty }{\Psi }^{\phi }({f}_{\alpha },\tau )\frac{1}{\sqrt{{f}_{\alpha }}}\phi (\frac{t-\tau }{{f}_{\alpha }})d\tau \frac{d{f}_{\alpha }}{{{f}_{\alpha }}^{2}},$$

where *C*_*φ*_ is a frequency scaling factor. A similar process can be applied to 2D seismic gather *D*(*r*, *t*), in which multi-dimension transformation is given by

5 $${\Psi }^{\phi }({f}_{\alpha },\tau ,{k}_{r})=\frac{1}{\sqrt{{f}_{\alpha }}}{\int }_{-\infty }^{\infty }D(r,t)\phi (\frac{t-\tau }{{f}_{\alpha }})dt{\int }_{0}^{R}{e}^{-i{k}_{r}r}dr$$

where *k*_*r*_ and *r* represent wavenumber and spatial distance, respectively, and Ψ^*φ*^(*f*_*α*_, *τ*, *k*_*r*_) denotes the 3D wavelet coefficients including times *τ*, frequencies *f*_*α*_, and wavenumbers *k*_*r*_.

### Step 1: Data-driven weight offset stacking

The design of stacking weights for the VS have rarely been fully studied in the existing literature (Zhao, *et al*.^21^). In contrast to previous works, this study proposes a fully data-driven weighting approach and wavelet domain implementation. The proposed new approach is formulated as

6 $${V}_{w({r}_{S})}({r}_{B}|{r}_{A};t)=\sum _{{r}_{S}}w({r}_{S})\{D({r}_{A}|{r}_{S};t)\otimes U({r}_{B}|{r}_{S};t)\}.$$

Our goal is to adaptively choose weighting coefficients *w*(*r*_*S*_) for all sources within the available offsets such that *V*_*w*_(*r*_*B*_|*r*_*A*_; *t*) can be computed with less contribution from non-stationary raypaths. The coefficients *w*_*rS*_ are entirely data-driven and directly computed from downgoing and upgoing wavefields. In order to evaluate the quality of individual *r*_*S*_ contributions to the retrieved *V*_*w*(*rS*)_(*r*_*B*_|*r*_*A*_; *t*), we use *V*(*r*_*B*_|*r*_*A*_; *t*) (Eq. (1)) to predict upgoing wavefields

7 $${U}_{pred}({r}_{B}|{r}_{S};t)=D({r}_{A}|{r}_{S};t)\ast V({r}_{B}|{r}_{A};t),$$

where * denotes convolution. Then we use the quality of the upgoing prediction *U*_*pred*_(*r*_*B*_|*r*_*S*_;*t*) to obtain weight coefficients. For time-lapse monitoring purposes, we customize Eq. (7) to be a target-oriented function focusing on the target reservoir. This function is designed to include a variety of seismic attributes such as *τ*, *f*_*α*_, *k*_*r*_ (TFK) of the target pre-stack events. Therefore we first perform transformation (3) for each upgoing trace Ψ^*φ*^(*f*_*α*_, *τ*) to obtain the 2D wavelet coefficients. To improve the robustness of the obtained weights, Eq. (5) is adapted with *U*_*pred*_(*r*_*B*_|*r*_*S*_; *t*) along the space dimension:

8 $${\Psi }_{{U}_{pred}}^{\phi }({f}_{\alpha },\tau ,{k}_{r})=\frac{1}{\sqrt{{f}_{\alpha }}}{\int }_{-\infty }^{\infty }U{}_{pred}({r}_{B}|{r}_{S};t)\phi (\frac{t-\tau }{{f}_{\alpha }})dt{\int }_{0}^{R}{e}^{-i{k}_{r}r}dr$$

The initial weighting coefficients is computed as follows

9 $${w}_{{r}_{S}}\bar{=}\sum _{{k}_{r}}\sum _{\tau }\sum _{{f}_{\alpha }}{w}_{{r}_{S}}({f}_{\alpha },\tau ,{k}_{r})=\sum _{{k}_{r}}\sum _{\tau }\sum _{{f}_{\alpha }}\frac{|{\Psi }_{U}^{\phi }({f}_{\alpha },\tau ,{k}_{r}){\Psi }_{{U}_{pred}}^{\phi }({f}_{\alpha },\tau ,{k}_{r})|}{|{\Psi }_{U}^{\phi }({f}_{\alpha },\tau ,{k}_{r})||{\Psi }_{{U}_{pred}}^{\phi }({f}_{\alpha },\tau ,{k}_{r})|}.$$

**Figure 3:**
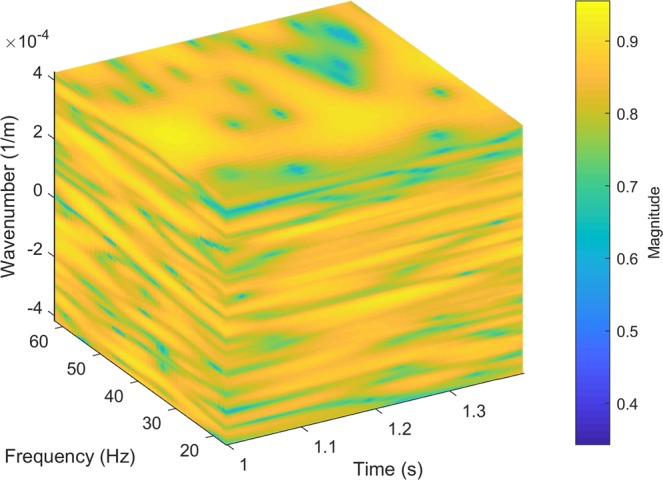
The 3D cross-coherence spectrum (display in log scale) of the TFK cube of the target reflections, computed via Eq. (5).

**Figure 4:**
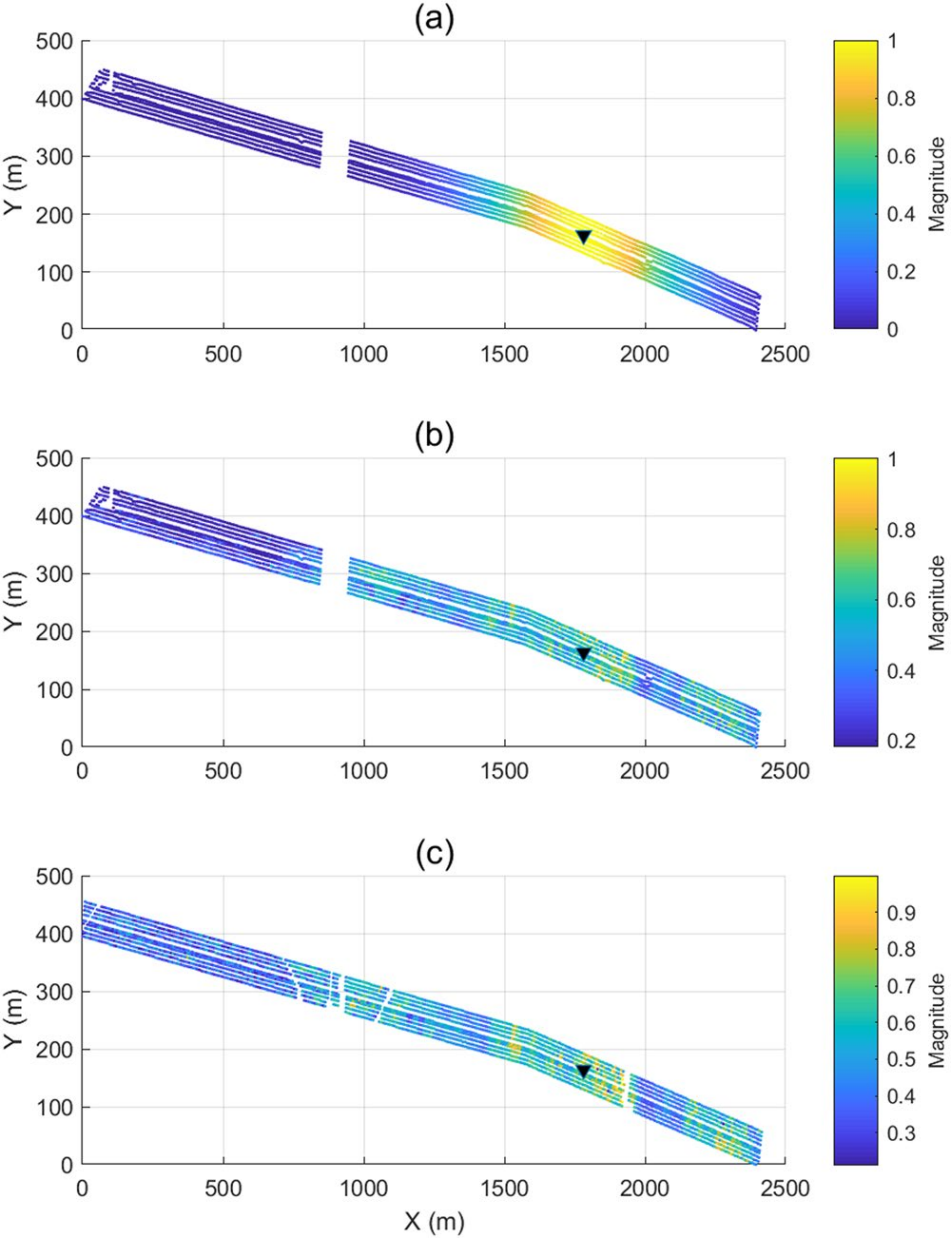
Stacking weights from 9 shot lines of surface sources that contribute to VS (#60) including those from (**a**) a conventional stacking operator of survey S1 and S11, (**b**) from the offset stack of survey S1, and (**c**) from the offset stack of survey S11.

**Figure 5:**
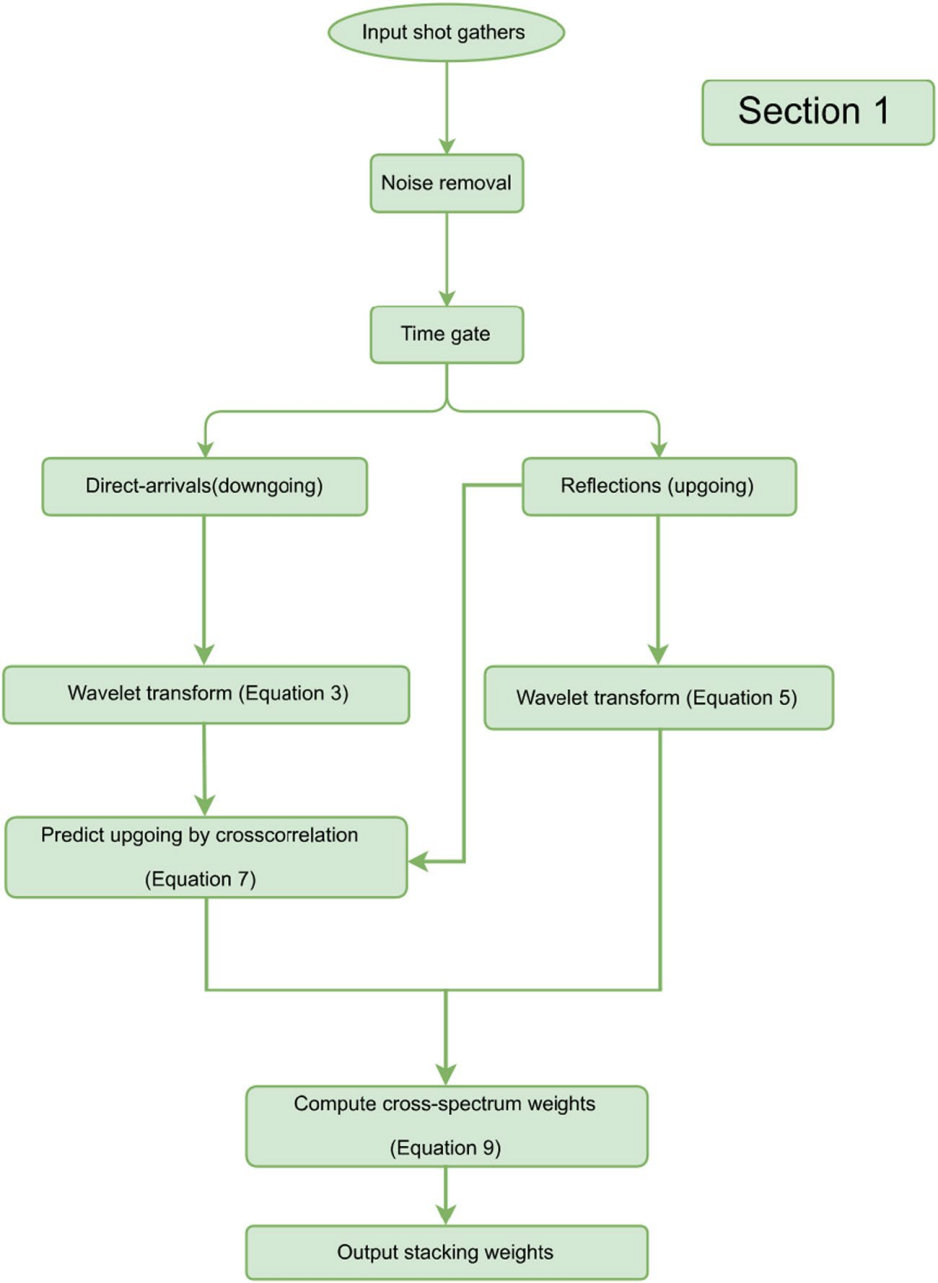
The summarized workflow is colored in green for the section of step 1. The input data is from the buried geophones, and the output comprises adaptive weights for VS offset stacking.

Equation (9) represents the normalized cross-spectrum *w*_*rS*_ between the predicted *U*_*pred*_(*r*_*B*_|*r*_*S*_) and the recorded *U*(*r*_*B*_|*r*_*S*_) in the TFK domain Figure 3 illustrates a cross-spectrum TFK cube from a selected event. We may restrict the range of ${\Psi }_{U}^{\phi }({f}_{\alpha },\tau ,{k}_{r})$ and ${\Psi }_{{U}_{pred}}^{\phi }({f}_{\alpha },\tau ,{k}_{r})$ to retain the target features, and mask other unassociated events. As discussed in the introduction section, the target horizon (2000 m) is at about 1 s–1.4 s, and has an effective bandwidth of about 20 to 60 Hz^1^. Therefore we limit the ranges of *τ* and *f*_*α*_, and sum over the corresponding frequencies, time lags, and wavenumbers to produce *w*_*rS*_. Equation (9) is then applied to these time-lapse datasets. The resulting stacking weights for the conventional and the new stacking are compared in Fig. 4. The offset stacking of survey S1 (Fig. 4b) and survey S11 (Fig. 4c) offers data-driven, and survey dependent weights, therefore, respond adaptively to the changes between times in the near-surface at a given VS. In contrast, the conventional spatial taper (Fig. 4a) only provides a static weight distribution for all surveys. Generally, the offset weights decrease with offset and tend to be smaller than the equivalent weights from the conventional taper. Among all surface sources to each of the selected VS locations, the offset weights show similar contribution patterns for different surveys, but also adapt to time-lapse changes shown in the minor pattern dissimilarity. It also can be observed that the near-offset shots have larger contributions. Due to the data-driven nature of the weighting coefficients, they may be influenced by noise and experience large lateral variations. A smoothing function can be applied to stabilize the data-driven stacking process. We summarize this section of step 1 in a workflow, colored as green, in Fig. 5.

### Step 2: Wavelet-crosscorrelation filtering for multi-wave mode contamination

Crosscorrelation can be used to determine the relative time delay between two seismic signals. The wavelet coefficients provide a local time and frequency distribution of the seismic traces. Equation (1) can be written in the form of the wavelet crosscorrelation^29^:

10 $$W{V}_{V({r}_{B}|{r}_{A})}({f}_{\alpha },\tau )=\sum _{{r}_{S}}g(r)\{\mathop{\mathrm{lim}}\limits_{T\to \infty }\frac{1}{T}{\int }_{-T/2}^{T/2}{\Psi }_{D({r}_{A}|{r}_{S})}^{\phi }({f}_{\alpha },t)\,{\Psi }_{U({r}_{B}|{r}_{S})}^{\phi }({f}_{\alpha },t+\tau )dt\},$$

where ${\Psi }_{D}^{\phi }({f}_{\alpha },t)$ and ${\Psi }_{U}^{\phi }({f}_{\alpha },t)$ are the wavelet coefficients of direct-downgoing *D*(*r*_*A*_|*r*_*S*_) and reflection-upgoing waves *U*(*r*_*B*_|*r*_*S*_), respectively. The wavelet crosscorrelation function *WV*_*V*(*rB*|*rA*)_(*f*_*α*_,*τ*) allows the detection of nonstationary coherence structures and the potential time-lag between two seismic traces.

It can be shown that *WV*(*f*_*α*_, *τ*)^26^ can be related to the classical crosscorrelation *V*(*r*_*B*_|*r*_*A*_) in the Fourier domain:

11 $$WV({f}_{\alpha },\omega )={f}_{\alpha }V(\omega ){|\phi ({f}_{\alpha }\omega )|}^{2},$$

12 $$|WV({f}_{\alpha },\omega )|={f}_{\alpha }|V(\omega )|{|\phi ({f}_{\alpha }\omega )|}^{2},\,{\rm{and}},$$

13 $$\angle WV({f}_{\alpha },\omega )=\angle V(\omega ),$$

where *WV*(*f*_*α*_, *ω*) is the wavelet crosscorrelation coefficient represented in the wavelet frequency and angular-frequency domain. *V*(*ω*) and *φ*(*f*_*α*_*ω*) denotes the cross-spectrum and mother wavelet in the Fourier domain, respectively. Equation (11) suggests that *WV*(*α*, *ω*) has the same phase spectrum as *V*(*ω*), and ∠ represents the phase spectrum. In Eq. (12), the amplitude spectrum |*WV*(*f*_*α*_, *ω*)| is the cross-spectrum |*V*(*ω*)| weighted by the factor *f*_*α*_|*φ*(*f*_*α*_*ω*)|^2^. Note that *WV*(*f*_*α*_, *ω*) is a complex-valued function. Subsequent filtering is applied to the amplitude spectrum only. The original phase of the recorded data is retained to honor the kinematics of the Green’s function extracted from the VS process.

The 2D TF domain filter, denoted as *H*(*f*_*α*_, *τ*), is applied to the obtained wavelet crosscorrelation *WV*_*i*_(*f*_*α*_, *τ*):

14 $$W{V}_{o}({f}_{\alpha },\tau )=H({f}_{\alpha },\tau ){\ast }_{\alpha ,\tau }W{V}_{i}({f}_{\alpha },\tau ),$$

**Figure 6:**
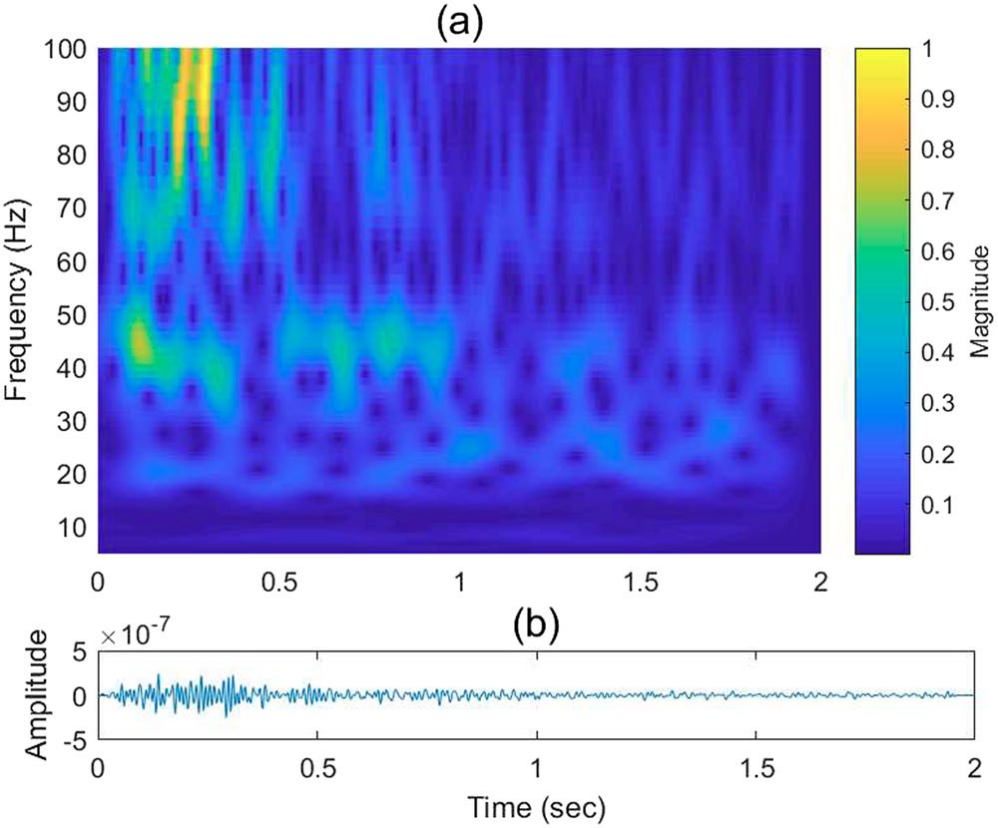
Field-data example showing (**a**) the wavelet coefficients of a VS trace in the TF domain via wavelet crosscorrelation and (**b**) the corresponding VS trace in the time domain.

**Figure 7:**
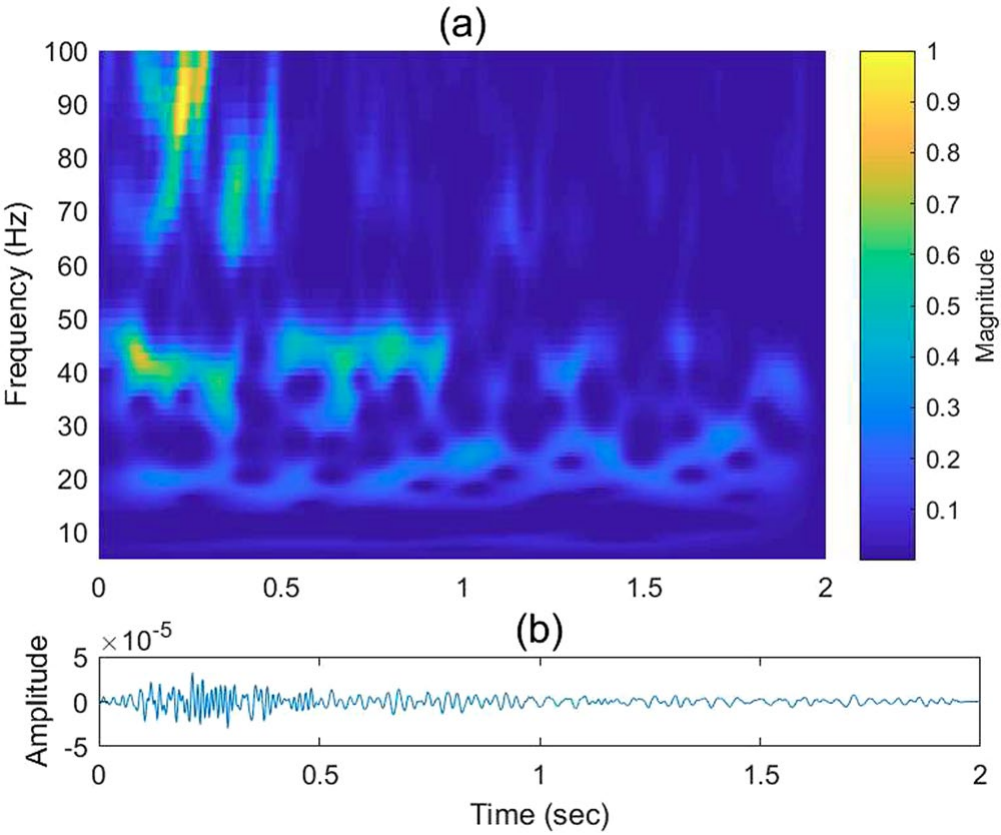
As shown in Fig. 6, but after soft-thresholding filtering of the amplitude spectrum in a sliding window.

Equation (14) denotes the 2D convolution operator, and *WV*_*o*_(*f*_*α*_, *τ*) is the output wavelet crosscorrelation in the TF domain. *H*(*f*_*α*_, *τ*) may take various 2D filter forms. For the discussion here, we use the first derivative of a 2D Gaussian filter as an example:

15 $$\begin{array}{rcl}H({f}_{\alpha },\tau ) & = & \frac{\partial {G}_{{f}_{\alpha }}}{\partial {f}_{\alpha }}\frac{\partial {G}_{\tau }}{\partial \tau }\\  & = & \{\frac{{f}_{\alpha }}{{\sigma }_{{f}_{\alpha }}^{2}}\exp \{-\frac{1}{2}[{(\frac{{f}_{\alpha }}{{\sigma }_{{f}_{\alpha }}})}^{2}+{(\frac{\tau }{{\sigma }_{\tau }})}^{2}]\}\{\frac{\tau }{{\sigma }_{\tau }^{2}}\exp \{-\frac{1}{2}[{(\frac{{f}_{\alpha }}{{\sigma }_{{f}_{\alpha }}})}^{2}+{(\frac{\tau }{{\sigma }_{\tau }})}^{2}]\}\\  & \mathop{=}\limits^{\Delta } & {H}_{{\sigma }_{{f}_{\alpha }}}({f}_{\alpha }){H}_{{\sigma }_{\tau }}(\tau ),\end{array}$$

where *σ*_*fα*_ and *σ*_*τ*_ are the standard deviations along *f*_*α*_,*τ* axes that parameterize the filter. Small *σ*_*fα*_ and *σ*_*τ*_ values lead to higher localization resolution along the *f*_*α*_,*τ* axes. The Gaussian filter can be replaced by other filters depending on the purpose of processing. As an example, the resulting wavelet coefficients before and after TF filtering are illustrated in Figs 6 and 7, respectively. The strong energy events (yellow-orange patches) represent the reflections generated by the target reflectors, whereas the weaker blue-green events with random shapes represent scattered noise. Removing the spurious events is desirable to enhance the overall S/N prior to correlation. Figure 7a demonstrates that *H*(*f*_*α*_, *τ*) strengthens these deep targets by suppressing the background noise. The inverted time series before and after *H*(*f*_*α*_, *τ*) filtering is shown in Figs 6b and 7b, respectively.

We extend 2D TF *H*(*f*_*α*_, *τ*) into 3D TFK *H*(*f*_*α*_, *τ*, *k*) filtering, which is expected to mitigate coherent noise in space/wavenumber dimension. Extending the 2D TF filtering given in Eq. (14), we propose a 3D filtering in the time-frequency-space (TFX) domain as

16 $$W{V}_{o}({f}_{\alpha },\tau ,x)=H({f}_{\alpha },\tau ,x){\ast }_{{f}_{\alpha },\tau }W{V}_{i}({f}_{\alpha },\tau ,x)$$

**Figure 8:**
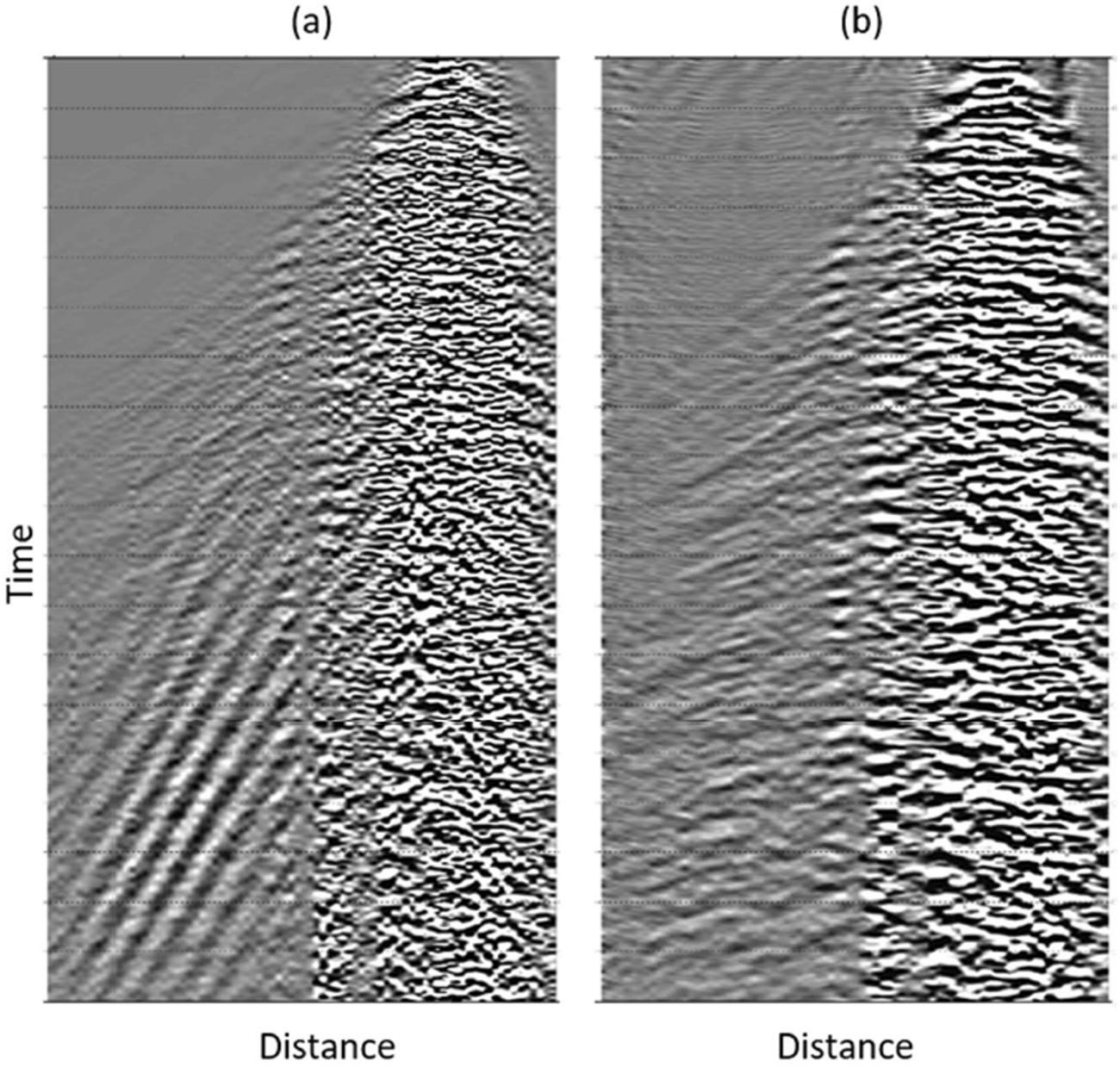
A VS shot gather obtained from (**a**) crosscorrelation and (**b**) wavelet crosscorrelation followed by TF and TFK filtering.

**Figure 9:**
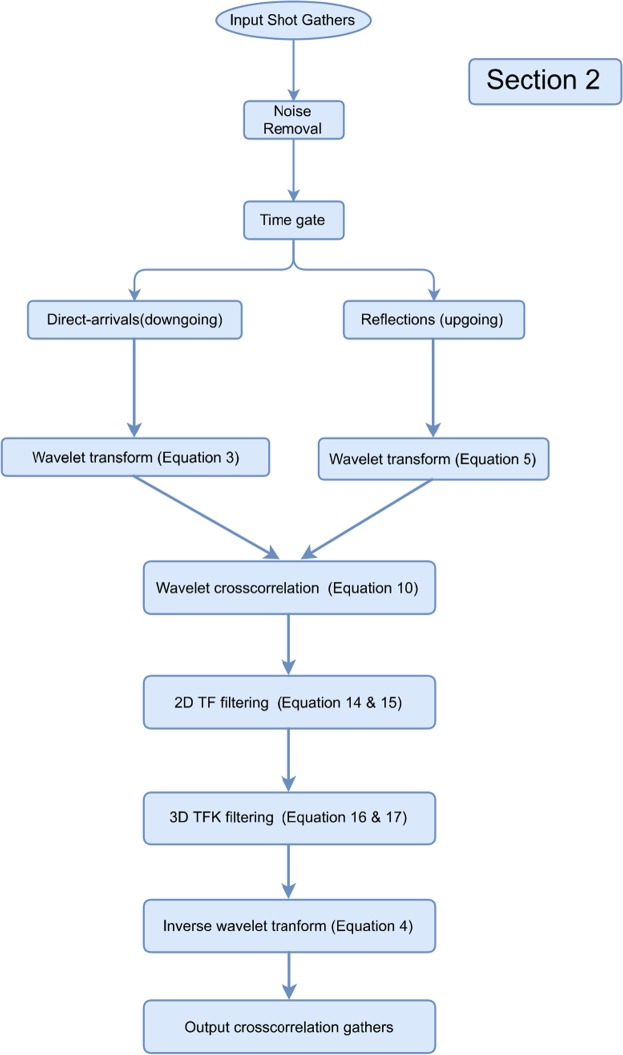
The summarized workflow is colored in blue for the section of step 2. The input data is from the buried geophones, and the output comprises crosscorrelation gather after wavelet filtering.

As a simple example, a 3D filter for *H*(*f*_*α*_, *τ*, *k*) can be expressed as:

17 $$\begin{array}{rcl}H({f}_{\alpha },\tau ,k) & = & \frac{\partial {G}_{{f}_{\alpha }}}{\partial \alpha }\frac{\partial {G}_{\tau }}{\partial \tau }{H}_{k\alpha }({f}_{\alpha },k)\\  & = & {H}_{{\sigma }_{{f}_{\alpha }}}({f}_{\alpha }){H}_{{\sigma }_{\tau }}(\tau ){H}_{k{f}_{\alpha }}({f}_{\alpha },k),\end{array}$$

where *H*_*kα*_(*f*_*α*_, *k*) can be a simple *f*_*α*_, *k* bandpass filter allowing a particular propagation velocity to pass while filtering out other wavefield components. As another example, we apply the multi-channel filter *H*(*f*_*α*_, *τ*, *k*) to process an entire VS shot gather contaminated by surface waves(Fig. 8a). A TFX cube is generated by gathering slices from each receiver in response to a common virtual shot to form the TFX cube. The data is then Fourier transformed along the receiver dimension to obtain the corresponding wavenumber spectra. Figure 8b shows the recovered signal after applying the inverse wavelet transform. Figure 8 demonstrates that the wavelet crosscorrelation was able to eliminate residual surface waves with a simple TFX filter, where the traditional VS failed. We summarize this section of step 2 in a workflow, colored in blue,in Fig. 9.

### Step 3: Radiation-pattern correction for unbalanced illumination

To address the unbalanced illumination issue, we used a method^30^ which iteratively constructs and applies a matched filter for an ideal 3D amplitude spectrum for direct downgoing arrivals. The simplest approach to estimate an isotropic radiation pattern is to compute the Green’s function of direct P-waves in a homogenous model^31^. This homogenous model is 2.5D and built independently for every VS, spanning from the buried receiver up to the surface with the radius consisting of the contributing sources. By targeting an optimal approximation to the direct P-wave arrivals before correlating with the reflections, the correct image associated with a cleaner direct P-wave can be enhanced, while near surface induced artifacts are suppressed. The following workflow describes radiation-pattern correction.

**Figure 10:**
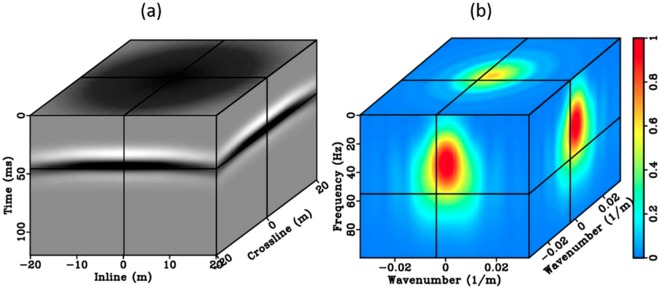
The target wavefield generated from the homogenous model (**a**) and its 3D FK spectrum (**b**).

Estimate the initial homogeneous model. The length of the straight ray connecting source and receiver is divided by the automatically picked onset arrivals. The output is the velocity estimate for each source-receiver pair. The local initial model above each VS is then derived as the average of the calculated velocities. Using this model, the synthetic direct arrivals are generated using an analytical 3D acoustic Green’s function^31^

18 $${D}_{P}({r}_{A}|{r}_{S};t)=\frac{\phi (t-r\chi )}{4\pi r}{e}^{-\frac{\omega t}{2Q}},$$

where *D*_*P*_(*r*_*A*_|*r*_*S*_; *t*) represents the output synthetic direct P-waves. *D*_*P*_(*r*_*A*_|*r*_*S*_; *t*) is then converted into FK domain ${\tilde{D}}_{P,{r}_{A}|{r}_{S}}({k}_{r},\omega )$, where *k*_*r*_ and *ω* are wavenumber and angular frequency, respectively. Tildes above the symbols indicate quantities in the FK domain. The FK amplitude spectrum of the field data ${\tilde{D}}_{{r}_{A}|{r}_{s}}({k}_{r},\omega )$ can be computed similarly*. φ*(*t* − *rχ*) still denotes the source Ricker wavelet (Eq. (3)), *r* is the distance between the surface source *r*_*S*_ to the buried VS *r*_*A*_ with the constant slowness *χ* (the inverse of P-wave velocity). 1*/*4*πr* is the geometrical spreading of a spherical wavefield in homogenous medium. ${e}^{-\frac{\omega t}{2Q}}$ represents the attenuation term of seismic amplitude of this analytical 3D acoustic Green’s function, where *Q* is a constant seismic quality factor derived from the data to match waveform amplitudes, and estimated from a nearby acoustic log. The dominant frequencyis 35 Hz to match the wavelet pattern of the target reservoir reflection. We solve Eq. (18) using the initial homogenous velocity, and illustrate the target syntheticresponsesin Fig. 10.

Estimate the matched filter so that the filtered recorded wavefields match with the modeled wavefields. A cost function in the FK domain is designed to shape the radiation pattern of the field data to match that of the modeled synthetic data as follows

19 $$J(\tilde{{\bf{H}}})=\mathop{{\rm{\min }}}\limits_{\tilde{{\bf{H}}}}{\Vert {\tilde{{\bf{D}}}}_{{{\bf{r}}}_{{\bf{A}}}}\tilde{{\bf{H}}}-{\tilde{{\bf{D}}}}_{{\bf{P}}{\boldsymbol{,}}{{\bf{r}}}_{{\bf{A}}}}\Vert }_{2}^{2},$$

where ${\tilde{{\bf{D}}}}_{{{\bf{r}}}_{{\bf{A}}}}$ and ${\tilde{{\bf{D}}}}_{{\bf{P}}{\boldsymbol{,}}{{\bf{r}}}_{{\bf{A}}}}$ are the vector representations (including contributing surface sources *r*_*S*_) of the 3D amplitude spectrum of ${\tilde{D}}_{{r}_{A}|{r}_{s}}({k}_{r},\omega )$ and ${\tilde{D}}_{P,{r}_{A}|{r}_{S}}({k}_{r},\omega )$ associated with the field data *D*(*r*_*A*_|*r*_*S*_; *t*) and the synthetic *D*_*P*_(*r*_*A*_|*r*_*S*_; *t*), respectively. The corresponding matched filter $\tilde{{\bf{H}}}$ is an L2 norm constrained least-square solution, and $J(\tilde{{\bf{H}}})\,$ is the corresponding cost function. We define $\tilde{{\bf{H}}}$ as vector multiplication in the FK domain, whereas the matched filter is traditionally defined as a convolution in the time-space (TX) domain. Since we operate on the amplitude spectrum and leave the phase intact, ${\tilde{{\bf{D}}}}_{{{\bf{r}}}_{{\bf{A}}}}$, ${\tilde{{\bf{D}}}}_{{\bf{P}}{\boldsymbol{,}}{{\bf{r}}}_{{\bf{A}}}}$, and $\tilde{{\bf{H}}}$ are real numbers. Noting that Eq. (19) is solved iteratively, ${\tilde{{\bf{D}}}}_{{\bf{P}}{\boldsymbol{,}}{{\bf{r}}}_{{\bf{A}}}}$ is also updated with a new velocity model, and therefore is a function of the matched filter from the previous iteration. $\tilde{{\bf{H}}}$ is updated iteratively using:

20 $${\tilde{{\bf{H}}}}_{{\bf{i}}+{\bf{1}}}={\tilde{{\bf{H}}}}_{{\bf{i}}}+{({\tilde{{\bf{D}}}}_{{{\bf{r}}}_{{\bf{A}}}}^{{\bf{T}}}{\tilde{{\bf{D}}}}_{{{\bf{r}}}_{{\bf{A}}}})}^{-1}{\tilde{{\bf{D}}}}_{{{\bf{r}}}_{{\bf{A}}}}^{{\bf{T}}}({\tilde{{\bf{D}}}}_{{\bf{P}}{\boldsymbol{,}}{{\bf{r}}}_{{\bf{A}}}}-{\tilde{{\bf{D}}}}_{{{\bf{r}}}_{{\bf{A}}}}{\tilde{{\bf{H}}}}_{{\bf{i}}}).$$

**Figure 11:**
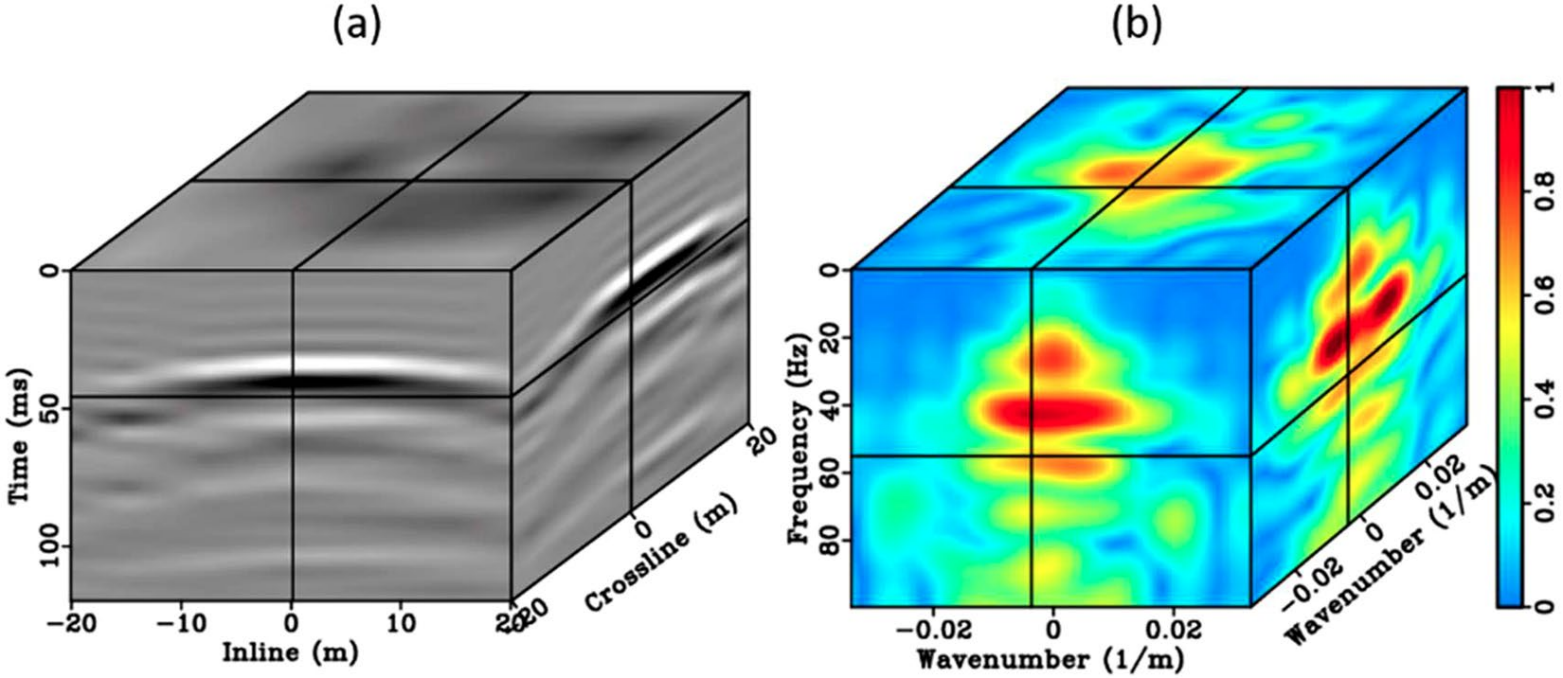
3D representation of the original direct wavefield (**a**) and its 3D FK spectrum (**b**).

This is an iterative process since ${\tilde{{\bf{D}}}}_{{\bf{P}}{\boldsymbol{,}}{{\bf{r}}}_{{\bf{A}}}}$ is also updated as a function of $\tilde{{\bf{H}}}$. The matched filter for the 3D amplitude spectrum ${\tilde{{\bf{D}}}}_{{{\bf{r}}}_{{\bf{A}}}}$ and ${\tilde{{\bf{D}}}}_{{\bf{P}}{\boldsymbol{,}}{{\bf{r}}}_{{\bf{A}}}}$ may be considered as a zero-phase FK filter, enforcing an isotropic radiation pattern associated with the desired direct P-wave, while leaving the phase intact. Since this field example has a good initial model estimation, only five iterations are needed for the inversion of the velocity and the matched filter to converge. The stopping criteriais the summation of the misfit less than 1*e*^−2^. Figure 11 illustrates the gated original direct arrivals of the 3D field common-receiver array (aperture 30 m at 7.5 m sampling interval).

Update the homogeneous model velocity. The homogeneous overburden model is iteratively updated, and coupled with the matched filter update. The velocity model is updated by solving the same minimization cost function, Eq. (20), but with the matched filter set to the value obtained from the previous iteration and optimized over slowness *χ*. After the initial model is built, the slowness can be updated iteratively

21 $$\begin{array}{rcl}{\chi }_{i+1} & = & {\chi }_{i}-\alpha \langle \nabla {{\bf{g}}}_{{\chi }_{i}}\rangle \\ \nabla {{\bf{g}}}_{{\chi }_{i}} & = & (\frac{\omega {\bf{r}}}{2Q}-\frac{\phi \text{'}{\boldsymbol{(}}{\bf{t}}-{\bf{r}}{\chi }_{i}{\boldsymbol{)}}{\bf{r}}}{\phi {\boldsymbol{(}}{\bf{t}}-{\bf{r}}{\chi }_{i}{\boldsymbol{)}}})\cdot {{\bf{F}}}_{t,r\to \omega ,{k}_{r}}^{-1}\{{\tilde{{\bf{e}}}}^{{\bf{j}}{\boldsymbol{\phi }}}[{\tilde{{\bf{D}}}}_{{\bf{P}}{\boldsymbol{,}}{{\bf{r}}}_{{\bf{A}}}}^{T}({\tilde{{\bf{D}}}}_{{{\bf{r}}}_{{\bf{A}}}}\tilde{{\bf{H}}}-{\tilde{{\bf{D}}}}_{{\bf{P}}{\boldsymbol{,}}{{\bf{r}}}_{{\bf{A}}}})]\},\end{array}$$

where *χ*_*i*_ is the constant slowness model, and ∇***g***_*χi*_ is the gradient for the *i*_*th*_ iteration. The FK phase term of the original direct wavefields ${\tilde{{\bf{e}}}}^{{\bf{j}}{\boldsymbol{\phi }}}$ is retained through the workflow, while the amplitude spectrum is iteratively updated with $\tilde{{\bf{H}}}$. An inverse multi-dimension Fourier transformation ${{\bf{F}}}_{t,r\to \omega ,{k}_{r}}^{-1}$ brings data from the FK to TX domain. *φ*(**t** − **r***χ*_*i*_) still denotes the Ricker source wavelets (Eq. (18)) in a vector notation (including all contributing surface sources) with respect to the VS. Since *χ*_*i*_ is a constant value, symbol 〈〉 simply averages the gradient ∇**g**_*χi*_ for all contributing sources. We adapt a linear search to determine the step length *α*, and *χ*_*i*_ is then iteratively updated in TX domain. An updated *χ*_*i*_ is plugged back to Eq. (18), and repeats until the iterations are finished.

Apply the estimated matched filter to direct wavefields and perform VS. With a few iterations, $\tilde{{\bf{H}}}$ is applied from Eq. (20)

22 $$\begin{array}{rcl}V({r}_{B}|{r}_{A};\omega ) & = & \sum _{{r}_{S}}g(r)\{{{\bf{D}}}^{h}({r}_{A}|{r}_{s};\omega ){\bf{U}}({r}_{B}|{r}_{s};\omega )\}\\  & = & \sum _{{r}_{S}}g(r)\{{({{\bf{F}}}_{r\to {k}_{r}}^{-1}\{{\tilde{{\bf{e}}}}^{{\bf{j}}{\boldsymbol{\phi }}}[{\tilde{{\bf{D}}}}_{{r}_{A}|{r}_{s}}\tilde{{\bf{H}}}]\})}^{h}\,{\bf{U}}({r}_{B}|{r}_{s};\omega )\}.\end{array}$$

**Figure 12:**
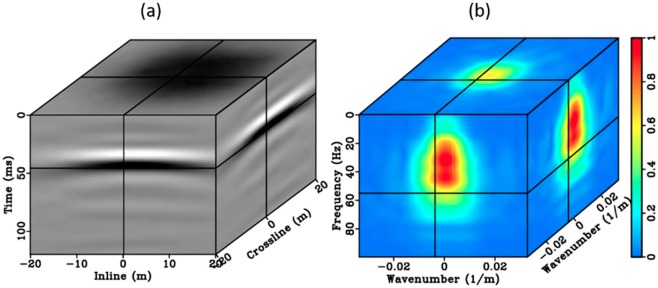
The inverted direct wavefields generated with the matched filter applied (**a**) and its FK spectrum (**b**).

**Figure 13:**
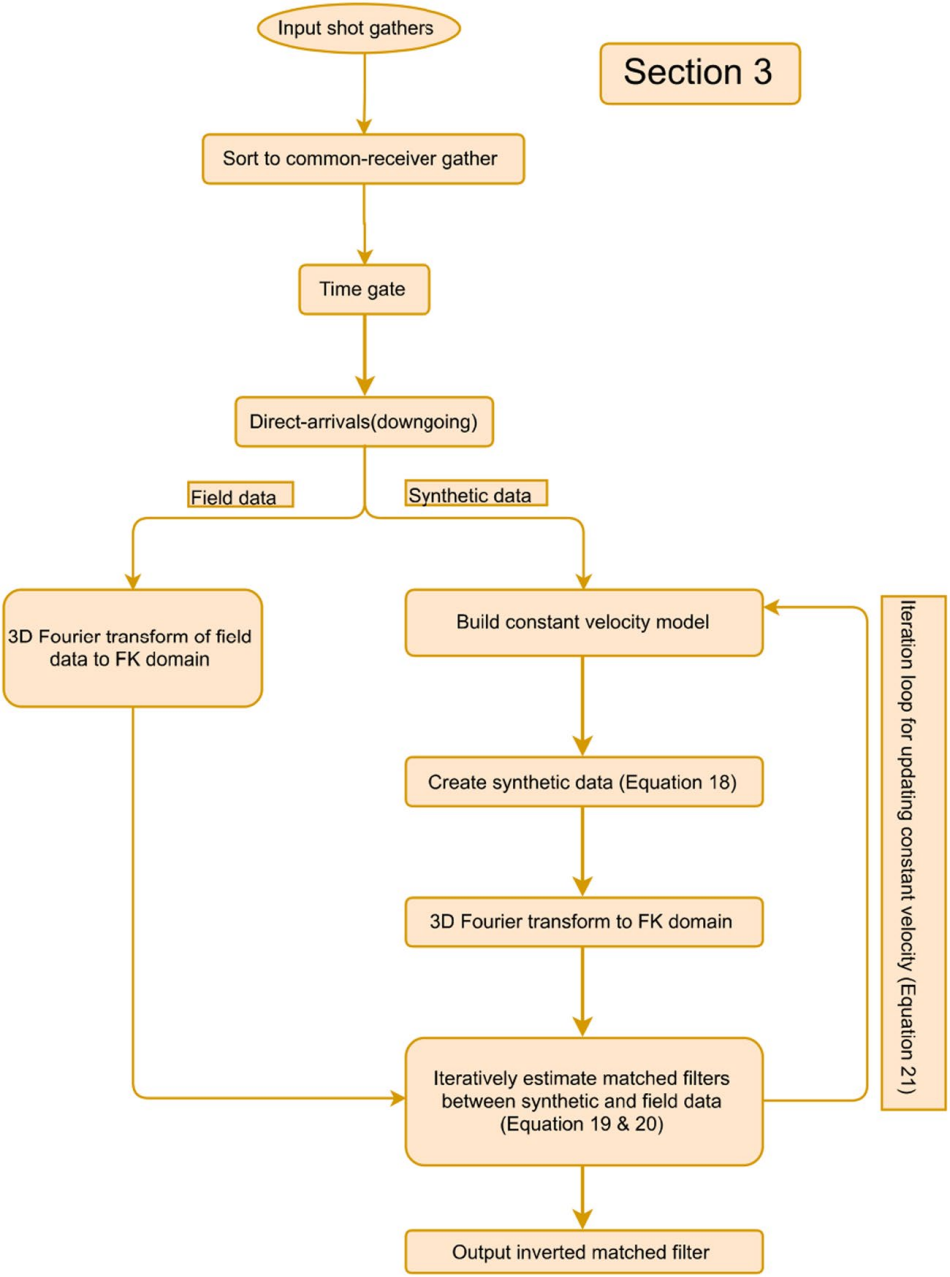
The summarized workflow is colored in orange for the section of step 3. The input data is from the buried geophones, and the output is comprised of an inverted matched filter after radiation-pattern correction.

*V*(*r*_*B*_|*r*_*A*_; *ω*) is the final output with radiation pattern correction, and a final Fourier transformation back to *V*(*r*_*B*_|*r*_*A*_; *t*). Again ${\tilde{{\bf{e}}}}^{{\bf{j}}{\boldsymbol{\phi }}}$ is isolated from the filter $\tilde{{\bf{H}}}$ to maintain its originality. The adaptively updated matched filter is only applied to the direct-downgoing wavefield prior to crosscorrelation with the upgoing wavefield. Ideally, the matched filter should not affect any time-lapse signal carried by the reflected wavefield after crosscorrelation Figure 12 is the matched filter $\tilde{{\bf{H}}}$ output after the final iteration. In this example, first-arrival picks provide velocity updates with sufficient quality so that the output from the matched filter after five iterations already provides reasonable results. In the time domain, the iteratively computed matched filter effectively regularizes spectrum bandwidth across the gathers, suppresses the coda waves, and stabilizes the wavelet character (Fig. 12). The direct-downgoing wavefields (Fig. 11) gradually approach the ideal P-wave (Fig. 10) associated with the isotropic radiation pattern. The reason we are proposing an iterative inversion theme, is that there is always some error associated with auto-pickers. We summarize this section of step 3 as an workflow (orange)in Fig. 13.

## Results

**Figure 14:**
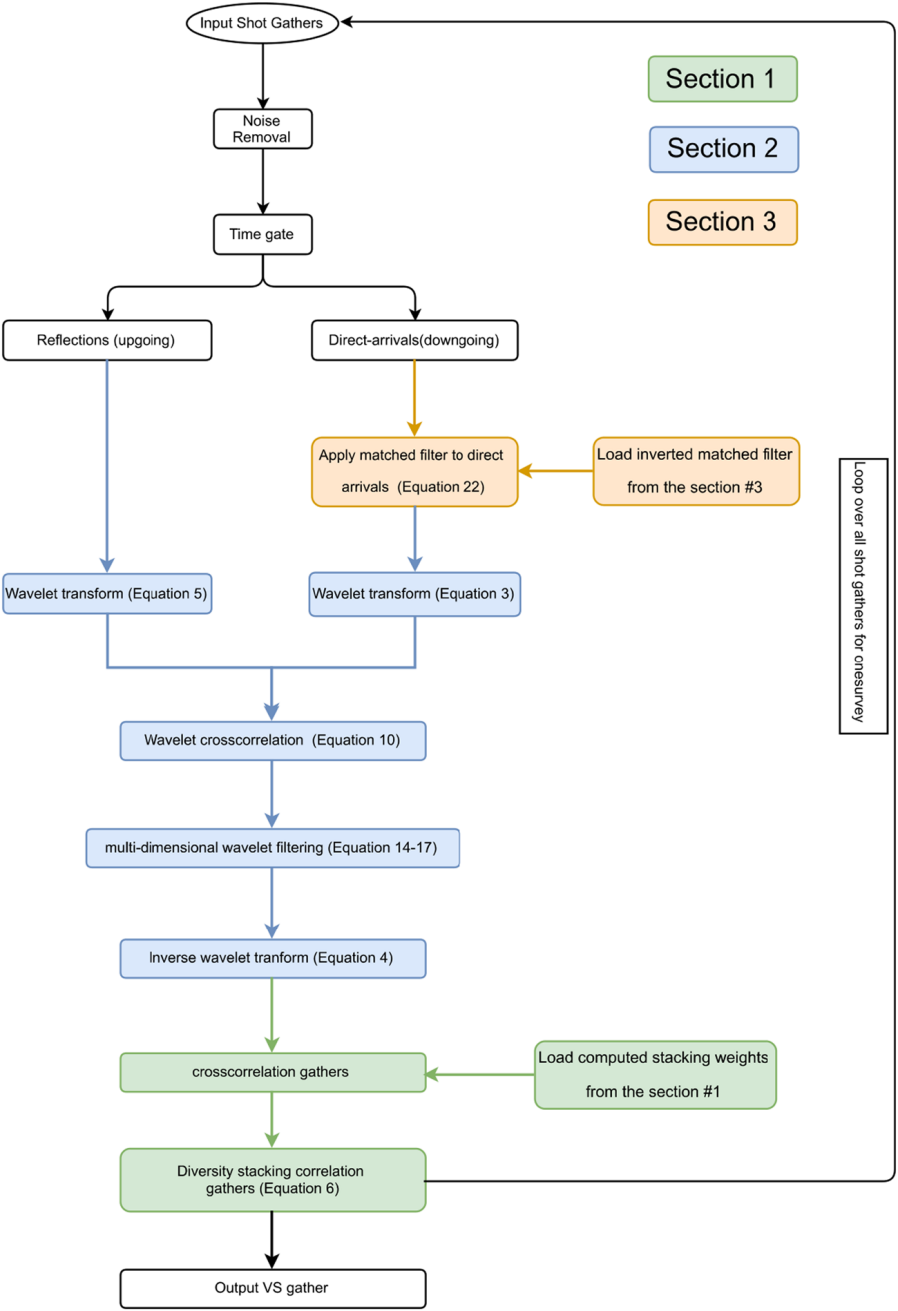
The overall workflow consist of step 1 (green), step 2 (blue), and step 3 (orange) for our comprehensive VS processing. The output VS gather combines all proposed three steps and to be used for subsequent imaging.

To assess the potential performance gain of this proposed workflow, the buried receiver data from the same series of field surveys was processed in three stages. These stages involved noise removal, VS redatuming, and CMP stacking. In this study we focus on the second stage, where four estimates of the proposed workflows were evaluated independently for each VS, including conventional procedures, using the offset stacking (the step 1), plus the wavelet-correlation filter (the steps 1 + 2), and the radiation-pattern correction (the steps 1 + 2 + 3). Figure 14 summarizes this proposed comprehensive workflow by organizing Fig. 5 (green), Fig. 9 (blue), and Fig. 13 (orange) in an orderly manner. During the VS processing, an auto-picking algorithm selected the first arrivals and windowed the direct-arrival energy in a 60 ms time window. All four outputs of each VS were obtained in parallel from post-correlation gathers stacked within common offsets. A non-VS CMP stack was created as well for comparison.

**Figure 15:**
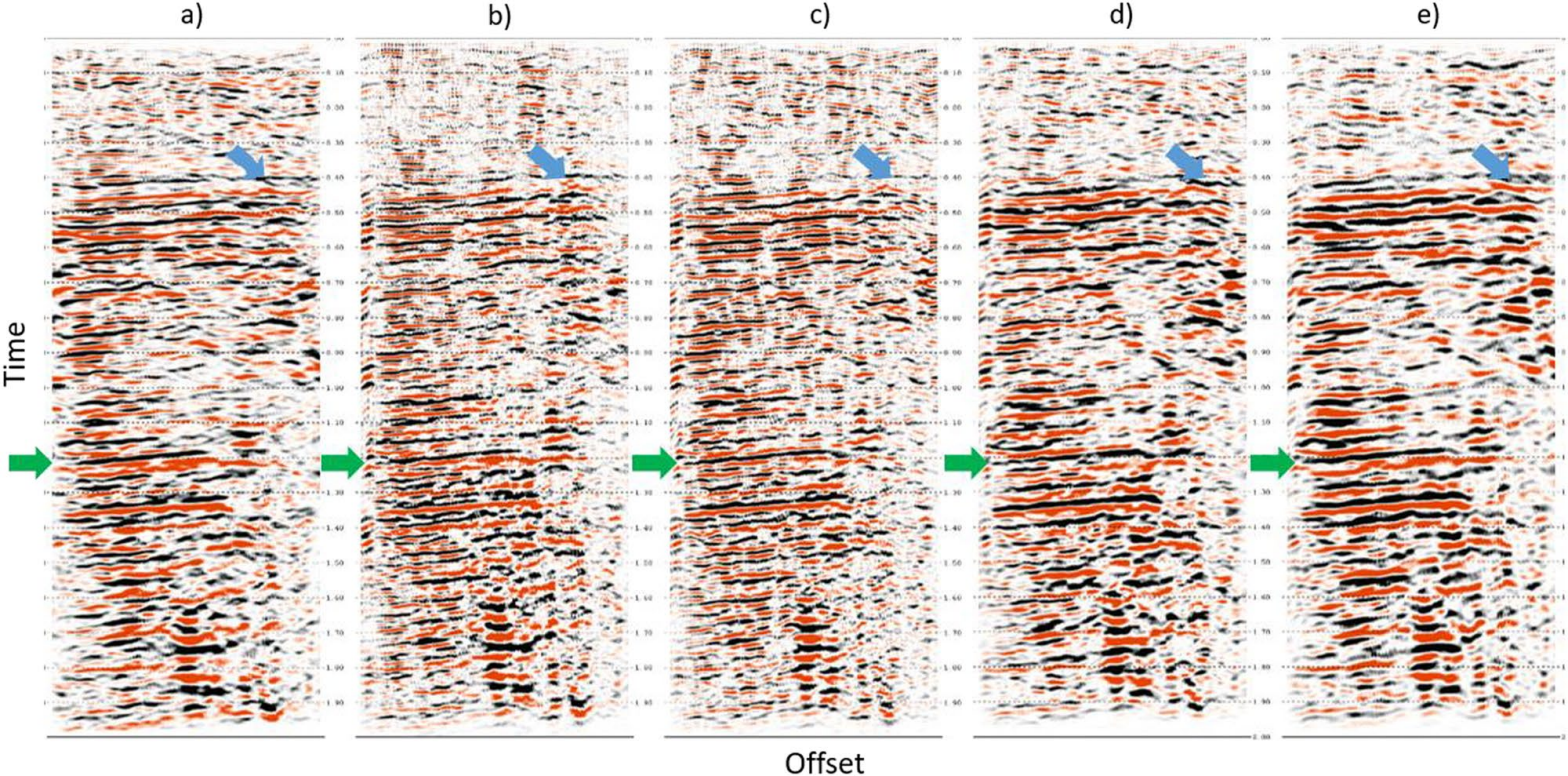
Survey 1 stacks obtained from (**a**) non-virtual source (non-VS), (**b**) conventional VS, (**c**) VS using the offset stack, (**d**) VS using the offset stack and the wavelet-crosscorrelation filtering, and (**e**) VS using the offset stack, the wavelet-crosscorrelation filtering and the radiation-pattern correction. The green and blue arrows indicate the SNR improvement on the target reflector and a selected shallow reflector, respectively. Panel (e) with the complete workflow has the best continuity of all five sections.

We plot the non-VS and proposed VS stacks of a selected survey in Fig. 15. The target horizon and selected areas are marked with green and blue arrows, respectively. The non-VS control section (Fig. 15a) shows good signal continuity on the marked reflectors, whereas the conventional VS stack (Fig. 15b) appears noisier with less reflection continuity in some zones. The VS stack (Fig. 15c) using the offset stack (step 1) shows better signal-to-noise ratio (SNR) and better continuity when compared with the conventional stack. Taken one step further, the VS stack (Fig. 15d), using the offset stack and the wavelet-correlation filter (the steps 1 + 2), demonstrates reduced contamination from the residual surface waves (steeply dipping, cross-cutting events) and scattering noise. Finally, the comprehensive VS stack (Fig. 15e), with the offset stack, the wavelet-correlation filter, and radiation-pattern correction (the steps 1 + 2 + 3), shows better SNR for the major reflectors.

**Figure 16:**
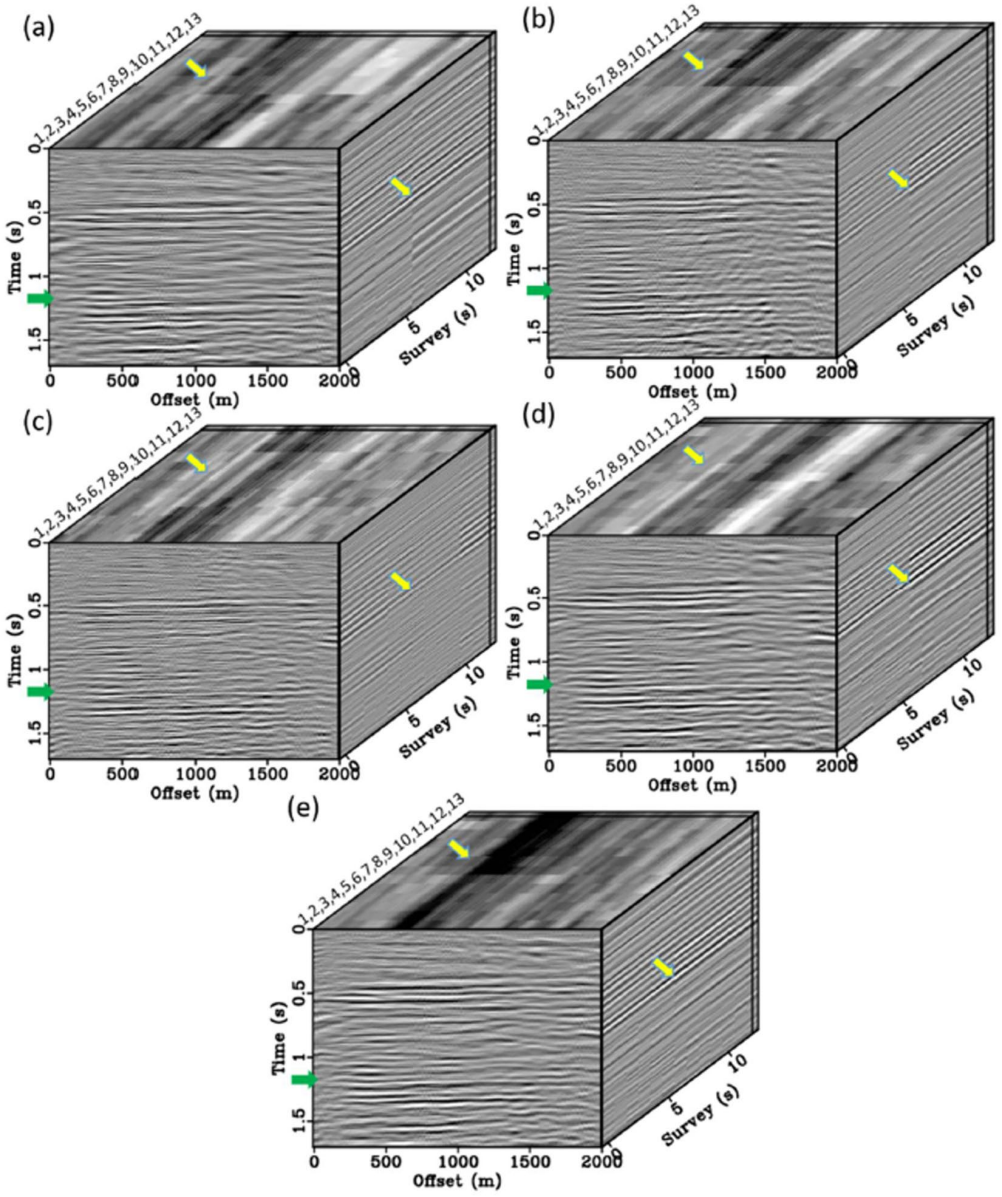
3D volume plots of 2D CDP stacks of all 13 surveys including (**a**) non-virtual source (non-VS), (**b**) conventional VS, (**c**) VS using the offset stack, (**d**) VS using the offset stack and the wavelet-crosscorrelation filtering, and (**e**) VS using the offset stack, the wavelet-crosscorrelation filtering and the radiation-pattern correction. The green arrows indicate the SNR improvement on the target reservoir. Discontinuity issue between surveys S1-S6 and surveys S7-S13 marked by the yellow arrows. Panel (e) shows the best continuity along the time-lapse dimension.

Figure 16 shows a 3D volume comprised of all 13 surveys of 2D stacks in terms of a repeatability illustration. Recall that there is no time-lapse signal in this data, so we are focusing on repeatability only. Specifically, we can compare surveys across time at specific sections (the arrows in Fig. 16). Similar repeatability issues can be observed from survey S6 to survey S7 in the non-VS control section (Fig. 16a), particularly at the areas marked by the yellow arrows. This gap is caused by the 17-month acquisition break. The conventional VS (Fig. 16b) shows repeatability improvement but the gap still exists. In contrast, we observe that using our comprehensive workflow gradually mitigates these discontinuities (Fig. 16c,d). Finally, Fig. 16e eliminates this break using a series of our proposed steps (steps 1 + 2 + 3). It shows a marked improvement in repeatability at all reflectors, especially for the time slices at the target level.

As we discussed in the step 1, the offset-stacking theme is a fully data-driven and target-oriented for the reservoir target. As a result, we observe that Fig. 16c strengthens the time-lapse continuity of the reservoir target from survey S6 to survey S7. As a side effect, however, other depths were subjected to experience a loss of time-lapse repeatability, especially the discontinuities around surveys 5–6 and 11–12. There should be other data-driven reasons associated with seismic properties. Specifically, as discussed in step 1, the target response of the monitored reservoir is around 1.0 s–1.4 s with an effective bandwidth of about 20 Hz–60 Hz. The data-driven weight is therefore adjusted to capture its response pattern in the TFK domain (Eq. 9). As a result, this algorithm strengthens the continuity of those events associated with the target TFK properties, whereas other events (e.g. the high-frequency or shallow events) were subjected to repeatability loss. Currently, we are in a preliminary stage of implementation where the stacking weights are determined in a heuristic way instead of through systematic optimization. A more robust and self-adaptive weights is one of the on-going research directions.

**Figure 17:**
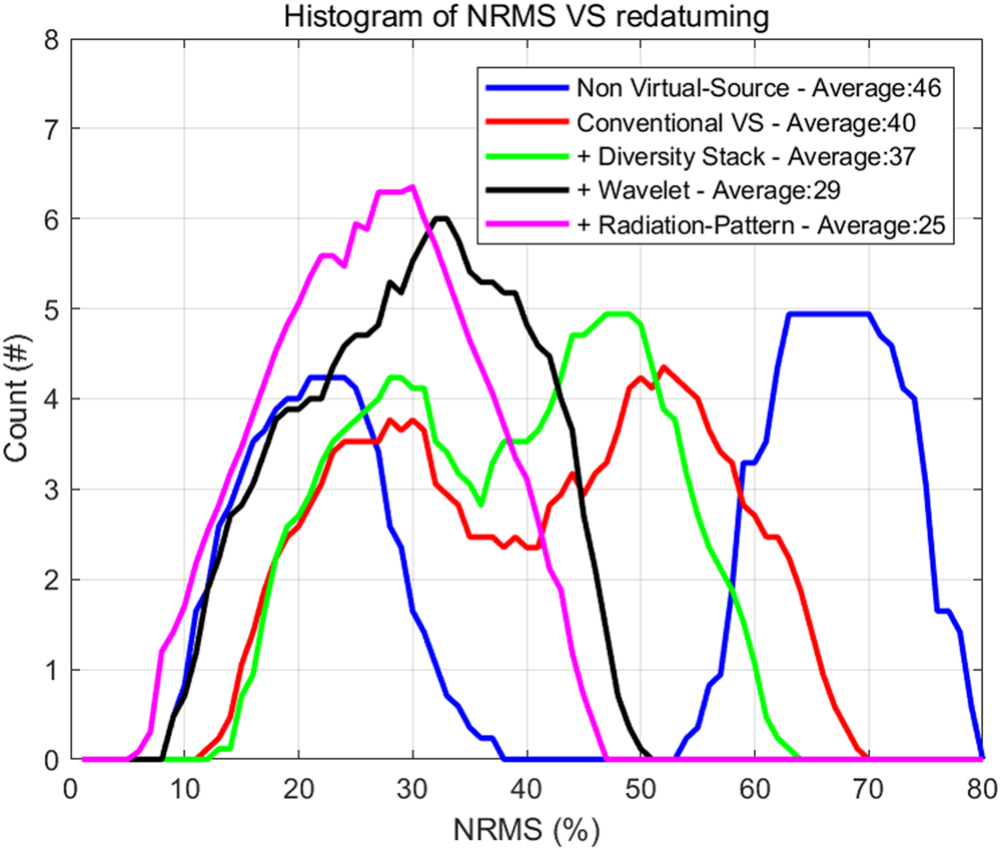
Histogram showing NRMS computed using the four processing flows. Median values of each distribution is displayed in the legend.

We use a normalized root mean square (NRMS) computed over a small time window around the target zone to quantify the repeatability^32^. This is typically calculated between two seismic traces in a given window, divided by their average RMS, expressed as a percentage. NMRS is sensitive to the differences in seismic waveforms and extremely sensitive to the smallest of changes in the data. NRMS is computed between all surveys, which results in 78 NRMS combinations at each common-depth point (CDP). A histogram of NRMS values for each method is displayed in Fig. 17. The non-VS control section (blue line) shows a bi-modal NRMS distribution (with peaks around 20% and 65%). Not surprisingly, the larger NRMS values are for surveys split between the two survey groups S1-S6 and S7-S13. A similar distribution, though narrower and less pronounced than the non-VS, is also observed for the conventional VS (red) and the offset stack VS (green). In contrast, the wavelet VS (black), using steps 1 + 2, bridge the bi-modal gap and result in a single peak value 29%. In the end, the combined process (workflow steps 1 + 2 + 3) produces the best NRMS values centered at about 25% (light blue). These results support the observations made from Fig. 16 and indicate that the proposed strategy can effectively reduce overburden effects between surveys separated by a 17 month gap, and an improvement from the conventional VS.

## Disucssion and Conclusion

We review three articles and summarize a comprehensive strategy to improve image quality and repeatability of virtual source redatuming in the presence of the complex near-surface. This VS method consists of a data-driven offset stack, wavelet-crosscorrelation, and radiation-pattern correction. In the first stage, the offset stack integrates wavelet transformation and cross-coherence analysis. The offset weight coefficients are directly computed from the data, based on the coherence similarity of the predicted upgoing wavefields and the original upgoing wavefields. The second step of the wavelet-crosscorrelation filtering integrates wavelet transformation, crosscorrelation, non-stationary TF, and TFK filtering. This integration maintains signal coherence across frequency and wavenumber bands, and exploits scale dependency at each frequency and wavenumber to allow for better noise filtering and signal separation. Effective noise suppression and high-resolution separation of nonstationary signals is achieved using TF, TFK, or TFX filtering of the wavelet-correlation coefficients. Lastly, prior to crosscorrelation, the direct arrivals are iteratively estimated using a zero-phase matched filter incorporating the 3D radiation pattern. This step modifies the radiation pattern of the virtual source, such that it closely resembles that of the ideal direct P-wave with isotropic radiation pattern. Iterative matched filtering effectively corrected the distorted radiation pattern of the direct-downgoing wavefields, and produced a significant improvement in illuminations.

Results from field data tests demonstrate that this comprehensive strategy can effectively attenuate virtual source artifacts, and produce distinct stack images without requiring a near-surface model. As time-lapse noise is mainly caused by near-surface variations over time, by reducing near-surface influences on reflection signals, the described methodology can improve time-lapse repeatability as well as imaging quality. In summary, offset stacking, crosscorrelation and denoising have been considered the three essential components of virtual source redatuming. Within this framework, we have demonstrated the value of our proposed workflows with a challenging onshore time-lapse application in a desert environment. Improvements were confirmed using the 13 time-lapse surveys that included a significant repeatability problem across a 17-month survey gap.

### Accession codes and data

Data & Code is available at: https://github.com/zhaoyangprof/waveletVS.git.
